# Research on the horizontal equity of inpatient benefits among NCMS enrollees in China: evidence from Shaanxi Province

**DOI:** 10.1186/s12913-018-3534-7

**Published:** 2018-09-19

**Authors:** Jue Yan, Yangling Ren, Zhongliang Zhou, Tiange Xu, Xiao Wang, Leilei Du, Yafei Si

**Affiliations:** 1 School of Public Policy and Administration, Xi’an Jiaotong University, No. 28 Xianning West Road, Xi’an, 710049 Shaanxi China; 2 International Business School Suzhou, Xi’an Jiaotong-Liverpool University, No. 111 Ren’ai Road, Suzhou, Jiangsu 215123 People’s Republic of China

**Keywords:** New rural cooperative medical system, Inpatient, Benefit equity, Concentration index, Decomposition of the concentration index

## Abstract

**Background:**

Equity is an important goal for countries in formulating relevant health policies, and research on the equity of health services is more important for China, where the gap between the rich and poor is widening. The aims of this study are to explore to what extent the benefit equity of New Rural Cooperative Medical System enrollees has been achieved and to determine the geographical disparities in Shaanxi province and thus provide suggestions for future policy formulations.

**Methods:**

Data were obtained from the fifth Health Service Survey of Shaanxi province in 2013. A two-step mode was used to analyse the influencing factors of the inpatient benefit rate and inpatient compensation fee. Concentration indexes and concentration curves were applied to measure the inequity of the inpatient benefit rate and inpatient compensation fee. The decomposition method was employed to explore the source of inequity and horizontal inequity.

**Results:**

Based on a sample of 38,032 enrollees, our results showed that there were pro-rich inequities in the inpatient benefit rate and compensation fee. The concentration index of the inpatient benefit rate and compensation fee in 2013 were 0.064 and 0.174, respectively. The economic level (224.62%), self-evaluated health status (− 25.89%) and occupation status (− 12.32%) were the primary three contributors to the inequity of the inpatient benefit rate, and the economic level (106.16%) and age (− 2.88%) were the first two contributors to the inequity of the compensation fee. There were regional differences in the sources of inequities. Moreover, pro-rich horizontal inequity remained after standardizing health care needs.

**Conclusions:**

Our results indicated that there were pro-rich inequities in the inpatient benefit rate and compensation fee in the New Rural Cooperative Medical System. The economic levels of enrollees accounted for most of the existing inequity, followed by self-evaluated health scores and age. Efforts should be made to strengthen policies and programmes in the New Rural Cooperative Medical System to achieve basic health services equity, such as implementing hierarchical medical treatments and reducing extra inpatient benefits for the rich.

## Background

Equity is an important goal as countries perfect relevant health policies. According to the universal health coverage proposed by the World Health Organization in 2013, a well-functioning health insurance system needs to meet both “depth” (the quantity and quality of medical services) and “height” (financial risk protection) conditions in addition to providing a wide range of coverage [1]. In China, the equity of health services has become increasingly more important with the widening of the gap between the rich and poor, and research in this area appears to be an exceedingly important topic.

The New Rural Cooperative Medical System (NCMS) is a basic medical insurance system established by the state to improve the health level of rural residents and solve the problem of “suffering from poverty and returning to poverty by illness”. The Chinese government began to readdress the issue of rural health care and launched the policy of NCMS in 2003 [2]. According to the 2003 Guideline, NCMS is a medical insurance model with government subsidies and has two important policy characteristics: the voluntary participation of the rural residents and the emphasis on preventing catastrophic diseases. The compensation model of NCMS is focused on health protection through risk pooling, which is on the basis of serious illnesses and supplemented with outpatients or minor illnesses. The institutional designs of NCMS are to provide medical subsidies to the participants, to alleviate the economic burden of disease on rural population, to solve the problem of peasants suffering from poverty due to serious illnesses, to improve access and equity of health care services in rural areas. The data suggest, as a rule of thumb, that the incidence of financial catastrophe will be negligible when out-of-pocket payments fall to or below 15–20% of total health expenditures [3, 4]. Last, the system can balance revenues and expenditures to maintain the sustainability of the insurance fund. By the end of 2013, 98.7% (8.02 billion) of the rural population was covered by the NCMS across the country, which is the health insurance program with the world’s largest coverage [5].

Regarding the current status of the NCMS, progress is relatively smooth and its coverage continues to improve, almost achieving the goal of “full coverage in 2010”. To some extent, the issue of rural residents’ medical treatment has been addressed and their economic burdens have been reduced. However, considerable problems and contradictions are highlighted with the development of the NCMS. The NCMS currently features a “low premium, high co-pay rate”. Whether rural residents use health services or not and what the utilization level will be mainly depends on the price of health care services. There is a lower probability of health care utilization with a higher co-payment and price. Some early studies have shown that people with low economic levels have greater flexibility in health services than people with high economic levels, and this high co-pay rate will make the rich who pay more use more health services and thus benefit more from them, implying that the rich will benefit more than the poor. The poor might subsidize the rich with the same level of payment in this case, to some extent, so that the benefits are unequal, which in turn, is contrary to the primary goal of the NCMS.

Therefore, based on the situation above, analysing the benefit level of insured NCMS residents and exploring the equity of inpatient benefits and the influencing factors of inequity are of significance to improve residents’ health and benefit status, even in a perfect policy. Some early studies have described the inequity situation in the NCMS. Qingfeng et al. showed that the poor need a large amount of health services, but the rich can gain more benefits than the poor from medical insurance [6]. Zhou et al. found that there were horizontal pro-rich inequities of the benefit rates and benefit degree [7]. Xiong et al. demonstrated that hospitalization costs, the number of days in bed due to illness, number of families’ nonagricultural workers population and regional policy differences were the most important factors that affected the equity of the NCMS [8]. Zhou et al. showed that there were pro-rich inequities of the HRQOL in both urban and rural China. Economic and educational statues were found to be two key factors in explaining the pro-rich inequity [9]. Liu et al. revealed that the NCMS was in favour of the rich, who can gain more compensation from the NCMS [10].

The NCMS mainly focuses on protecting inpatients from catastrophic expenditures from hospital compensations, but outpatient compensation is relatively limited. Hence, this research only focuses on the inpatient benefit of NCMS enrollees [11–13].

Broad-sense benefits include the increased use of health services, compensation and reduced disease burden; narrow-sense benefits mainly refer to compensation from the NCMS. Indicators, including the general outpatient treatment rate, number of visits, number of hospitalizations and hospitalization rate, are used to describe the residents’ utilization of health services. Compensation can additionally be explained in two aspects: the benefit rate and degree of benefit. This paper analyses the beneficiaries of the NCMS from the perspective of narrow-sense benefits and compensation [14].

From the economic perspective, equity refers to a fundamental criterion for allocating social resources, which means treating various groups with a reasonable and normative standard, despite distinctions between different groups regarding economy and interest. Accordingly, equity in healthcare includes the equity of health status, the equity of health service utilization, the equity of health financing and the equity of health resources allocation. Specifically, equity in the field of health service utilization embodied in the principle of on-demand distribution, which means that health care should be allocated according to health needs. Every member of society with health service needs should have the same opportunity to access appropriate healthcare, gender, race, age, place of residence or purchasing power should not be factors influencing such opportunities [15]. Therefore, if health care utilization is negatively correlated with health care needs, the inequality in healthcare utilization is regarded as inequity [16]. The equity of health service utilization is divided into horizontal equity and vertical equity. Horizontal equity requires that the same health services should be provided to people with the same health care needs, regardless of their socioeconomic status [17]. Additionally, vertical equity is specific to each individual, individuals with different health needs should be treated differently [18]. For example, people with higher needs for healthcare ought to have more access to healthcare.

The purpose of implementing the NCMS is to cope with the high medical fees and difficulties of attaining medical service, which aims to improve the use of health services, mitigate the economic burden and decrease the inequity of health use caused by income to a minimum. A good policy can be evaluated from the aspects of both equity and effectiveness. Equity should ensure that residents with the same needs attain equal services; effectiveness should improve medical treatment for residents. From the perspective of the benefits of the NCMS, residents should have the same compensation opportunities if they have the same medical needs and they should be able to enjoy the same level of compensation fees on the basis of compensation.

In this study, we investigated to what extent the benefit equity of NCMS enrollees has been achieved and the existing geographical disparities in Shaanxi province, which was also determined in other provinces with similar situations in China. An understanding of the nuanced geographical variations and crucial causes of inpatient benefit and compensation inequity is the first step toward reducing equities [19]. Furthermore, this study adopted an individual economic indicator (per capita household consumption expenditure) that can indicate the level of the benefit inequity more effectively, divided by the economic levels of NCMS enrollees [20]. The results of this study will be meaningful for advocacy, policy and programming for reducing inequities in Shaanxi and other provinces with similar situations in China.

## Methods

### Data source

Data were obtained from the fifth National Health Service Survey (NHSS) in Shaanxi province in 2013. The NHSS was organized and conducted by the Centre for Health Statistics and Information of the Chinese Ministry of Health. The main aims of this survey were to collect information on health care needs, utilization, quality and expenditures; to evaluate the performance of health reform and development from the previous five years; and to provide evidence for China’s health care planning, targets and action plans [21–24].

Representative samples were obtained through a four-stage stratified random sampling procedure before any individual interviews were conducted [25]. In the first stage, 32 out of the total of 107 counties or districts of Shaanxi Province were selected randomly as the preliminary sample. In the second stage, five townships or streets were chosen randomly from each of the selected counties or districts. In the third stage, two villages or residents’ communities in each selected township were selected randomly. In the last stage, 60 households were selected randomly in each sampled village/community. Finally, 20,700 households were identified.

The NHSS questionnaire mainly includes household demographics, socioeconomic status, self-reported health status and health care utilization. Data were collected from 57,529 respondents. The adult in-person response rate was 82.1%. Quality control was implemented throughout the survey. The investigators were mainly local medical workers, and the investigation instructors were doctors mainly from township hospitals or health institutions and above. The investigation instructors revisited 5% of the sample households and asked 14 key questions to determine the accuracy of the data recorded by the investigators. The compliance rate after the counter check was more than 97.7%. Various survey data tests, including Myer’s index, goodness-of-fit test, DELTA dissimilarity coefficient and GINI concentration ratio, were applied to check the quality and consistency of the data. The results showed that the survey had no age preference and that the size of the surveyed households was consistent with that of the entire local population.

For the purpose of this study, 38,032 samples of NCMS enrollees of age 15 years old and above were selected from the fifth National Health Service Survey in Shaanxi province in 2013.

### Description of variables

#### Measuring variables

Owing to the reality that the NCMS fund that is mainly used for inpatient compensation and outpatient compensation is rather limited, we selected the following two variables as the main measuring indexes in this study.

Inpatient benefit rate: The proportion of enrollees obtaining inpatient compensation from the NCMS during the last year. This rate signifies the probability of obtaining hospitalization compensation.

Compensation fee per capita: The amount of the inpatient compensation fee received from the NCMS on the basis of inpatient compensation over the past year. This variable reflects the degree of inpatient benefits.

#### Independent variables

In this study, the factors that affect the hospitalization benefit of the insured include demographic and sociological factors are as follows: Gender, age, marital status, degree of education, occupation, family size, health status (illness in last two weeks, chronic diseases and self-evaluated health), living region, economic level, and access to the nearest medical institution. Among them, gender, age, illness in last two weeks, chronic diseases and self-evaluated health were categorized as need variables; the others were categorized as nonneed variables. (A detailed description of the independent variables is presented in Table 1):

**Table 1:** Description of the independent variables and sociodemographic characteristics of NHSS enrollees

Independent variables	Explanation	Variable	Number	Percentage (%)
Need variables				
Gender		Male	18,435	48.47
(Baseline: Male)	Female = 1, male = 0	Female	19,597	51.53
Age		15–44	16,435	43.21
(Baseline: 15–44)	45 to 59 = 1, other = 0	45–59	12,716	33.44
	More than 59 = 1, other =0	> 59	8881	23.35
Nonneed variables				
Marital status		Single	4824	12.69
(Baseline: Single)	Married = 1, other = 0	Married	29,979	78.84
	Divorced, widowed or other = 1, other = 0	Divorced, widowed or other	3220	8.47
Degree of education		Primary or below	16,966	44.62
(Baseline: Primary or below)	Junior = 1, other = 0	Junior	15,654	41.17
	High or above = 1, other = 0	High or above	5405	14.21
Occupation (Baseline: Employed)		Employed	29,906	78.65
	Retired = 1,other = 0	Retired	296	0.78
	Student = 1, other = 0	Student	1708	4.49
	Unemployed = 1, other = 0	Unemployed	6112	16.07
Family size	The number of household registration and non-domicile permanent population	0–2	6375	16.76
		> 2	31,657	83.24

### Two-step model

The empirical research model used in this study contained two steps. We first inspected the factors that affect the insured’s capability of acquiring hospital compensation, which signifies the occurrence of hospital benefits. Subsequently, the factors that affect compensation fees are examined when the hospitalization benefit occurs. There exist hierarchical levels of this dataset. In our study, city was level 3 unit, county or district was level 2 unit and individual was level 3 unit. Therefore, we used multilevel modeling to analyze the inpatient benefit rate and inpatient compensation fee.

### Decomposition of the concentration index

The concentration index can be decomposed in two different ways: by component and by population subgroup. In this study, the concentration index of the hospital benefit was decomposed as the contribution of inequity by every single factor. Variables were divided into need variables and nonneed variables to calculate the horizontal inequity index. Need variables refer to the definition of benefit equity, while nonneed variables are irrelevant to benefit equity; missing them in the model would cause an offset of the regression coefficient of the need variables. The need variables include gender, age, illness in last two weeks, chronic disease and self-evaluated health. We use the generalized linear model and probit model to decompose the inequity of inpatient benefits and compensation fees among NCMS enrollees. The decomposition of the concentration index in this model is specified as below:

$$ {y}_i={\alpha}^m+\sum \limits_j{\beta_j}^m{x}_{ji}+\sum \limits_k{\gamma_k}^m{z}_{ki}+{\mu}_i $$

In the model above, *y*_*i*_ represents inpatient benefit; *x*_*ji*_ represents need variables; *z*_*ki*_represents nonneed variables; *β*_*j*_ and *γ*_*k*_ equal to partial regression coefficient, respectively; and *μ*_*i*_ denotes the error term. The result is specified as below:

$$ C=\sum \limits_j\left({\beta_j}^m{\overline{x}}_j/\mu \right){C}_j+\sum \limits_k\left({\gamma_k}^m{\overline{z}}_k/\mu \right){C}_k+{GC}_{\mu }/\mu $$

In the model above, C denotes the nonstandardized concentration index; *μ* is the mean value of *y*; ${\overline{x}}_j$ and ${\overline{z}}_k$ represent the mean values of need variables and non-need variables, respectively; *C*_*j*_ and *C*_*k*_ denote the concentration index of need variables and nonneed variables, respectively; and *GC*_*μ*_ captures the error term of the concentration index. Based on the result of this model, we can observe the degrees and directions of each factor’s effect on the inequity of inpatient benefits. For an independent variable, the contribution rate is equal to the percentage of the contribution value of the inpatient benefit concentration index. If the contribution value is positive, it signifies that the independent variable increases the pro-rich inequity of inpatient benefits, and vice versa. Some nonobserved variables that we named error terms were not included in this model; the contribution of the error terms is equal to the concentration index minus the sum of all known independent variables’ contributions. We can also calculate the horizontal inequity index of inpatient benefits, which is the result of the nonstandardized concentration index minus the inequity contribution value of the need variables.

## Results

Table 1 presents the sociodemographic characteristics of NHSS enrollees. Among NCMS enrollees, we included 38,032 samples aged 15 years old and above from three regions of Shaanxi province. Among them, 48.47% were males and 51.53% were females; the sex ratio was 1:0.94. enrollees aged 15–44 years accounted for 43.21%, 45–59 years accounted for 33.44%, and 59 years and above accounted for 23.35%. Among them, married individuals accounted for the largest proportion, at 78.84%. The education degree of primary school or below had the largest proportion of 44.62%, followed by enrollees with junior-level degrees (41.17%), and finally, those with degrees from high school or above accounted for a proportion of 14.21%. Among them, 78.65% were employed, 16.07% were unemployed, and 0.78% were retired. Family size was measured by the adjusted family size; those with a family size of two or more people accounted for a larger proportion (83.24%), while those with family members of less than two accounted for 16.76%.

The concentration index of the inpatient benefit of NCMS enrollees in different regions is presented in Table 2. For the full sample, according to the concentration index of the inpatient benefit rate, there were inequities favouring the rich in Guanzhong (0.0659) and Shannan (0.1033). On the contrary, the concentration index of the inpatient benefit rate in Shanbei was negative (− 0.0117), implying an inequity benefiting the poor. In addition, comparing the absolute values of the concentration indexes, we found the highest value in Shannan, implying that the equity of the inpatient benefit rate might be worst in this area.

**Table 2:** Concentration index of the inpatient benefit of NCMS enrollees in different regions

Living region	Concentration index	S.E.	95% CI	
			Lower	Upper
Benefit rate				
Shaanxi province	0.0639	0.0098	0.0446	0.0831
Shanbei	−0.0117	0.0221	− 0.0550	0.0316
Guanzhong	0.0659	0.0151	0.0364	0.0954
Shannan	0.1033	0.0161	0.0716	0.1349
Compensation fee				
Shaanxi province	0.1735	0.0167	0.1407	0.2062
Shanbei	0.2091	0.0396	0.1312	0.2868
Guanzhong	0.1571	0.0263	0.1056	0.2086
Shannan	0.1570	0.0251	0.1078	0.2063

Regarding the concentration index of the inpatient compensation fee, the concentration index in every region was positive: Shanbei (0.2091), Guanzhong (0.1571) and Shannan (0.1570), implying that there is an inequity slope to the rich in receiving compensation fees in these three areas; the richer the enrollees were, the more they received. When comparing the absolute value of the concentration index of the inpatient compensation fee in the three regions, the largest value in Shanbei implied that enrollees there might have the lowest equity of inpatient compensation fees.

The decomposition of the inpatient benefit rate inequity and compensation fee inequity of NCMS enrollees is shown in Table 3. The known variable contributed 173.09% to the inequity of the inpatient benefit rate, and the residual contribution rate was − 73.09%. Among the variables, the most significant contributors to the inequity of inpatient benefit were economic factors, self-evaluated health scores, occupation status and age. The economic factor contributed most to the inequity of the inpatient benefit rate of enrollees, with a contribution rate of 224.62% and a contribution value of 0.1691, indicating that higher-income enrollees benefited more. Moreover, the self-evaluated health scores contributed to the inequity of the inpatient benefit rate with a contribution rate of − 25.89% and a contribution value of − 0.0286, indicating that the self-rated health scores increased the inpatient benefit rate inequity slope to the poor. Occupation status and age contributed to the inequity of the inpatient benefit with contribution rates of − 12.32% and − 12.18%, respectively, and had negative contribution values, indicating that they affected the inequity of the inpatient benefit rate inclining to the poor.

**Table 3:** Decomposition of inpatient benefit rate inequity and inpatient compensation fee inequity of NCMS enrollees

Variable	Inpatient benefit rate				Inpatient compensation fee			
	Elastic coefficient	Concentration index	Contribution	Contribution rate (%)	Elastic coefficient	Concentration index	Contribution	Contribution rate (%)
Gender(Baseline: Male)								
Female	0.1388	0.0013	0.0017	2.2998	−0.1713	0.0049	− 0.0008	− 0.4689
Age (Baseline: 15–44)				−12.1817				−2.8799
45–59	−0.0731	0.0000	0.0000	0.0074	0.0712	0.0576	0.0041	2.2937
> 59	0.0499	−0.0185	− 0.0092	−12.2561	0.0819	− 0.1130	− 0.0093	−5.1736
Marital status (Baseline: Single)				1.0619				0.5240
Married	0.7377	0.0011	0.0080	10.5500	−0.4509	0.0152	− 0.0069	−3.8334
Divorced, widowed or other	0.0471	−0.0152	− 0.0071	−9.4853	− 0.0797	−0.0978	0.0078	4.3574
Degree of education (Baseline: Primary or below)				1.4255				0.8672
Junior	0.0017	0.0068	0.0001	0.1513	−0.0107	0.0807	−0.0009	− 0.4824
High or above	0.0050	0.0193	0.0010	1.2742	0.0120	0.2004	0.0024	1.3496
Occupation (Baseline: Employed)				−12.3229				−0.4259
Retired	0.0026	0.0027	0.0001	0.0933	−0.0033	0.0571	−0.0002	− 0.1052
Student	−0.0262	0.0197	−0.0052	−6.8503	0.0000	0.2476	0.0000	0.0065
Unemployed	0.0382	−0.011	− 0.0042	−5.5659	0.0106	− 0.0552	− 0.0006	− 0.3272
Living region (Baseline: Shannan)				−3.2764				1.7243
Guanzhong	−0.0477	− 0.0023	0.0011	1.4510	−0.0106	− 0.0251	0.0003	0.1486
Shanbei	−0.0237	0.0150	−0.0036	−4.7274	0.0359	0.0785	0.0028	1.5757
Illness in last two weeks (Baseline: Not ill)								
Ill	0.1071	0.0019	0.0020	2.6956	−0.0155	0.0090	−0.0001	− 0.0779
Chronic disease (Baseline: No chronic disease)								
Chronic disease	0.1943	−0.0027	− 0.0053	−7.0009	− 0.0415	− 0.0163	0.0007	0.3793
Self-evaluated health (Baseline: 0–59)				−25.8898				0.9878
60–79	−0.1091	− 0.0083	0.0091	12.0769	−0.1138	− 0.0267	0.0030	1.7011
80–100	−0.9741	0.0029	−0.0286	−37.9961	−0.2644	0.0048	−0.0013	−0.0013
Adjusted family size	−0.0169	− 0.0001	0.0000	0.0295	0.1946	−0.0050	−0.001	− 0.5451
Economic level	3.7812	0.0045	0.1691	224.6225	4.1506	0.0457	0.1899	106.1636
Go to the nearest medical institution (Minutes)	−0.0117	−0.0105	0.0012	1.6303	0.0456	−0.0773	−0.0035	−1.9708
R.E.				−73.0934				−4.2777
Sum				100				100

It was observed from the decomposition results that the contribution rate of the known variables to the inequity of inpatient compensation fee was 104.28% and that the residual contribution rate was − 4.28%. The factors that contributed more to inequity included economic levels and age. The contribution rate of the economic level was 106.16%, and its contribution value was positive, indicating that the economic level increased the benefit inequity of the compensation fee to the rich, which enabled the rich to receive more compensation fees. The contribution rate of age was − 2.88% with a negative contribution value, suggesting that age increased the inequity in favour of the poor.

The decomposition of inpatient benefit rate inequity and compensation fee inequity of NCMS enrollees in different regions is presented in Table 4. For inpatient benefit rate inequity, the most significant contributors to the inequity of insured inpatient benefits in Shanbei were he economic level (299.89%), illness in last two weeks (− 121.94%) and self-evaluated health scores (− 33.65%). In Guanzhong, the most significant contributors were the economic level (187.70%), self-evaluated health scores (− 11.69%) and occupation status (− 11.05%). In Shannan, the most significant contributors were the economic level (169.96%), self-evaluated health scores (− 20.01%), and age (− 7.54%). Regarding compensation fee inequity, we also found that the most significant contributors to the inequity of the insured inpatient benefit in Shanbei were the economic level (116.52%), age (− 7.95%) and self-evaluated health scores (− 6.74%). In Guanzhong, the most significant contributors were economic level (106.96%) and occupation status (− 12.26%). In Shannan, the most significant contributors were economic level (102.82%) and age (− 6.58%). In the three regions, the contribution values of the economic level were positive, indicating that the economic level increased the pro-rich inequity of inpatient benefits. The contribution values of the other variables were negative, implying that those factors increased the pro-poor inequity.

**Table 4:** Decomposition of inpatient benefit rate inequity and inpatient compensation fee inequity for NCMS enrollees in different regions

Variables	Inpatient benefit rate						Inpatient compensation fee					
	Shanbei		Guanzhong		Shannan		Shanbei		Guanzhong		Shannan	
	Contribution	Contribution rate (%)	Contribution	Contribution rate (%)	Contribution	Contribution rate (%)	Contribution	Contribution rate (%)	Contribution	Contribution rate (%)	Contribution	Contribution rate (%)
Gender(Baseline: Male)												
Female	0.0016	25.7279	0.0023	2.9907	0.0012	1.0301	−0.0057	−2.5082	−0.0029	− 2.9197	0.0014	0.9094
Age (Baseline: 15–44)		1.5503		−5.6397		−7.5351		−7.9522		−1.6436		−6.5781
45–59	0.0001	1.5503	0.0011	1.4950	−0.0015	−4.4235	0.0034	1.4963	−0.0010	−1.0468	0.0032	2.0185
> 59	−0.0381	0.0000	−0.0055	−7.1347	− 0.0036	−3.1116	− 0.0214	−9.4485	−0.0006	− 0.5995	−0.0137	−8.5966
Marital status (Baseline: Single)		−32.1578		2.2808		0.3334		2.5745		3.6204		2.7694
Married	0.0007	11.6937	0.0063	8.2154	0.0119	10.4714	−0.008	−0.4748	− 0.0046	−4.609	− 0.0024	−1.5339
Divorced, widowed or other	− 0.0027	−43.8516	− 0.0045	−5.9346	− 0.0116	− 10.1381	0.0069	3.0493	0.0081	8.2294	0.0069	4.3033
Degree of education (Baseline: Primary or below)		−12.8425		4.5601		−1.3953		3.2369		−4.6464		−1.4492
Junior	−0.0004	−5.6382	0.0002	0.2219	0.0013	−0.1378	0.0003	0.1524	−0.0018	−1.802	−0.0004	− 0.2366
High or above	−0.0004	−7.2043	0.0033	4.3382	−0.0014	−1.2575	0.0070	3.0845	−0.0028	−2.8444	−0.0019	−1.2126
Occupation (Baseline: Employed)		− 23.0369		−11.0455		−5.456		7.7825		−12.2559		2.0137
Retired	0.0000	0.0798	0.0000	−0.0086	0.0005	0.4218	0.0004	3.9741	−0.0000	−0.0050	− 0.0001	0.9880
Student	−0.0009	−14.0549	−0.0049	−6.4221	− 0.0039	−3.4037	0.0062	2.7466	−0.0098	−9.9497	0.0011	0.6603
Unemployed	−0.0006	−9.0618	−0.0035	−4.6148	− 0.0028	−2.4740	0.0024	1.0618	−0.0023	−2.3012	0.0006	0.3654
Illness in last two weeks (Baseline: Not ill)												
Ill	−0.0076	−121.9352	0.0043	5.5987	0.0038	3.2940	0.0018	0.7871	−0.0012	−1.1852	0.0002	0.1147
Chronic disease (Baseline: No chronic disease)												
Chronic disease	−0.0090	− 143.1894	− 0.0019	−2.4771	− 0.0038	−3.3669	−0.0025	− 1.0841	0.0000	0.0061	0.0017	1.0761
Self-evaluated health (Baseline: 0–59)		−33.6532		−11.6894		−20.0083		−6.7428		−2.3977		−2.2418
60–79	0.0035	5.5815	0.0053	6.9269	0.0112	9.8450	0.0148	6.5528	0.0028	2.8416	0.0011	−0.7750
80–100	−0.0266	−42.5727	−0.0141	−18.4167	− 0.0340	−29.8533	−0.0301	−13.2956	− 0.0047	− 4.7603	−0.0023	−1.4668
Adjusted family size	0.0021	3.3380	−0.0002	−0.1996	0.0004	0.3465	0.0104	4.6181	−0.0005	−0.4790	− 0.0002	−0.0970
Economic level	0.0188	299.8931	0.1436	187.6952	0.1937	169.9613	0.2636	116.5156	0.1020	103.1974	0.1639	102.8223
Go to the nearest medical institution (Minutes)	0.0022	34.8038	−0.0033	−4.2858	− 0.0021	−1.8770	−0.0045	−2.0053	− 0.0009	−0.9107	− 0.0046	− 2.8578
R.E.		−41.6876		−67.7885		−44.257		−4.8651		19.6143		1.2765
Sum	−0.0573	100	0.1285	100	0.1592	100	0.2450	100	0.0798	100	0.1545	100

**Fig. 1:**
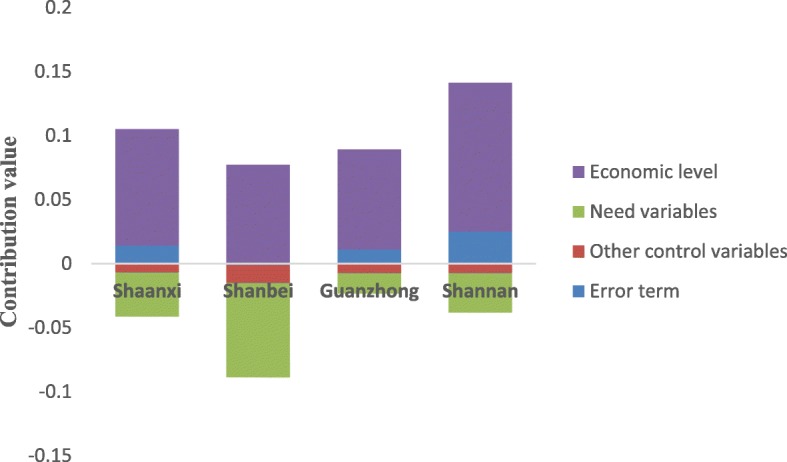
Classification analysis of the inpatient benefit rate inequity contribution for NCMS enrollees

**Fig. 2:**
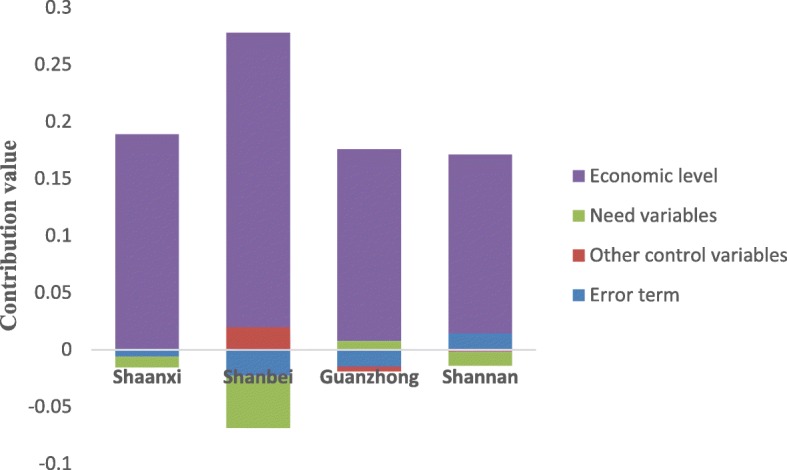
Classification analysis of the inpatient compensation fee inequity contribution for NCMS enrollees

The contributions of the economic level to the inpatient benefit rate inequity of NCMS enrollees in Shaanxi, Shanbei, Shannan and Guanzhong were above the level of the horizontal equity line, which implied that it increased the pro-rich inequity of the inpatient benefit rate; the contributions of need variables and other control variables were below the level of the horizontal equity line, which indicated that it increased pro-poor inequity (Fig. 1). It should be noted that the results of the classification analysis for the inequity contribution of the inpatient compensation fee in NCMS enrollees were consistent with that without other control variables (Fig. 2). It appeared that the contributions of the other control variables to the inequity of inpatient compensation fee in NCMS enrollees in Shaanxi and Shanbei were above the level of the horizontal equity line, indicating the existence of pro-rich inequity; to the contrary, the contributions in Shannan and Guanzhong were below the level of the horizontal equity line, indicating the existence of pro-poor inequity (Fig. 2). In addition, economic level was the most important factor contributing to both inpatient benefit rate inequity and compensation fee inequity for insured residents in Shaanxi, Shanbei, Guanzhong and Shannan.

Table 5 presents the horizontal inequity of the inpatient benefit of NCMS enrollees. It should be noted that the horizontal inequity indices of the inpatient benefit rate of the enrollees in Shaanxi province, as well as Shanbei, Guanzhong, and Shannan regions, were 0.0983, 0.0622, 0.0818 and 0.1333, respectively. The horizontal inequity indices were positive, indicating that there was inequity to the rich in the rate of inpatient benefit, which means that the rich received more hospital reimbursements than the poor in the face of the same need for health services. It was also observed that the horizontal inequity indices of the inpatient compensation fee in Shaanxi province, as well as the Shanbei, Guanzhong and Shannan regions, were 0.1827, 0.2550, 0.1493 and 0.1691, respectively. The horizontal inequity indices were positive, indicating that horizontal inequity existed in compensation fee reception, especially for the rich; the rich received more hospital reimbursements than the poor with the same need for health services.

**Table 5:** Horizontal inequity of inpatient benefit for NCMS enrollees

Equity index	Shaanxi province	Shanbei	Guanzhong	Shannan
Benefit rate				
Concentration index	0.0639	−0.0117	0.0659	0.1033
Contribution of needed variables	−0.0344	−0.0739	− 0.0159	−0.0305
Horizontal inequity index	0.0983	0.0622	0.0818	0.1338
Compensation fee				
Concentration index	0.1735	0.2091	0.1571	0.157
Contribution of needed variables	−0.0092	−0.0459	0.0078	−0.0121
Horizontal inequity index	0.1827	0.255	0.1493	0.1691

## Discussion

The data used in this study were obtained from the fifth National Health Service Survey (NHSS) in Shaanxi province in 2013. The survey was conducted by stratified cluster sampling and indicated sufficient representativeness after the Marye Index test. Regarding the education level, the proportion of enrollees with primary school education or below (44.62%) and junior high school education (41.17%) accounted for the overwhelming majority of enrollees. Only a small number of enrollees (14.21%) had a high school education or above, which was lower than the proportion of rural residents with a senior high school education or above in 2013 (15.40%), implying a relatively low education level of NCMS enrollees.

This study also found that women had higher rates of inpatient benefits than men (the sex ratio was 0.94:1) for at least two reasons. First, the relatively poor health status of women prompted their use of hospital services [26]. Women are subjected to both work and family pressure in a contemporary society, which gradually causes damage to their physical and psychological health. Diseases such as breast cancer, cervical cancer and others emerged more frequently in women in recent years, leading to more use of hospital services and thus benefits from NCMS health insurance. Second, women are also hospitalized for normal childbirth delivery, thereby further increasing the rate of inpatient benefits. The rate of inpatient benefits for enrollees aged 45–59 was slightly lower than that of enrollees aged 15–44, and the rate of inpatient benefits for enrollees over 59 was higher than that of residents aged 15–44. Compared with the 15–44-year-old population, enrollees over 59 years had a poorer health status and physical fitness, thus contributing to their use of hospital services and more benefits; the 45–59-year-old population was healthier than the 59-year-old population. In addition, their views and values were more conservative and economical. Thus, for some diseases requiring hospitalization, more outpatient treatment or self-treatment with fewer costs would be a primary choice for them, resulting in the lower rate of inpatient benefits.

We also found an interesting phenomenon: compared to the married, divorced or widowed enrollees, unmarried enrollees mostly aged between 15 to 25 years old maintained good health, used fewer hospital services and had a lower inpatient benefit rate.

The inpatient benefit rate of enrollees who were ill in the last two weeks was higher than those who were not, and the rate of inpatient benefit of enrollees with a chronic illness was higher than those who did not have chronic illnesses. The two-week prevalence of disease and the prevalence of chronic diseases can represent the health status of the sample population to some extent. The poor health status of fragile enrollees made their use of hospital services more expensive, leading to a higher rate of hospitalization, which is consistent with most of the previous findings [20, 27, 28]. The rate of inpatient benefits for self-evaluated health scores of 60–79 and 80–100 were lower than that of self-evaluated enrollees with a score of 0–59. The self-evaluated health score is a personal assessment of individual’s own health status. The self-evaluated health score is relatively stable and does not change rapidly with physical health in the short term. Largely the self-evaluated health score is a reasonable representation of a person’s health level. Generally, higher self-evaluated health scores represent better health conditions. According to our study, enrollees with lower self-evaluated scores had a higher rate of inpatient benefits; the economic level and the inpatient benefit rate were positively correlated. The gap between the rich and poor has continued to grow since the implementation of reform and opening up of China, along with the improvement of economic levels. Currently, to avoid the excessive use of health resources, China is setting a definite pay line, capping line and compensation ratio in the NCMS compensation policy. Only the portion of the reimbursement line below the payoff line and above the capping line is reimbursed at a certain percentage, and the part below the payoff line requires that patients pay for themselves. The state has made some adjustments to increase the proportion of reimbursement and the ceiling line. Some groups still abandon hospitalization but choose relatively low outpatient treatments or self-treatment because of excessive self-payments. Therefore, as for hospitalization needs, enrollees with higher economic levels have the financial basis to afford out-of-pocket inpatient expenditures, while enrollees with lower economic levels have to choose self-care or lower-cost outpatient clinics because of economic limitations, leading to lower inpatient benefit rates for the less-privileged population.

Another interesting finding was that women received fewer inpatient compensation fees compared with men. One speculation might be that a large proportion of female inpatients were hospitalized for pregnancy, and their hospitalization expenditures were lower than those of other diseases, so less compensation was obtained. Moreover, ideological differences caused by gender differences encourage women to be thriftier in spending money. In the situation of an illness, relatively conservative treatment was preferred by women, which made them less likely to be hospitalized.

Inpatient compensation fees were found to be positively related to age. Compared to 15–44 year olds, the inpatient compensation fee was higher for people aged 45–59 and 59 and above. A number of studies have shown that age has a significant effect on the compensation fee under the same conditions and that the compensation fee for older persons is generally higher [29–33]. This increase in the compensation fee with age is associated with the physiological structure of the human body, as various body indicators start to become worse with age; younger individuals tend to be more easily cured of identical diseases than older individuals. This feature leads to high expenditures in hospitalization for the elderly, resulting in more inpatient compensation to the elderly according to China’s NCMS compensation policy.

The results also showed that NCMS enrollees living in Guanzhong received fewer inpatient compensation fees than those dwelling in Shannan. One explanation for this might be that the rich who live in a more developed area always choose relatively good treatment options or higher levels of hospitalization, resulting in a high-level total expenditure of hospitalization and inpatient compensation fees. Our finding is consistent with most prior studies, where the inpatient compensation fee is positively associated with the socioeconomic level of enrollees, as the rich benefited more from the NCMS [11, 30–35].

We found that there was inequity sloped to the rich in the inpatient benefit rates and compensation fees; the incidence of hospitalization was more concentrated on the population with higher economic levels. Owing to the current compensation model, which was based on “actual medical expenses” in accordance with a certain ratio to be reimbursed, the rich received more inpatient compensation fees. We also found that the inequity in inpatient compensation fees was greater than the inequity in inpatient benefit rates by comparing their concentration indices, implying that the absolute value of the concentration indexes of the inpatient compensation fee was larger than the inpatient benefit rate in Shaannxi province and the other three regions. In addition, we found that Shanbei had the best horizontal equity of the inpatient benefit rate and Shannan had the worst, while Guangzhong had the best horizontal equity of the inpatient compensation fee and Shanbei had the lowest. It is likely that the different physical conditions caused by the geographic environments and economic development of NCMS enrollees may have contributed to the differences in horizontal equity. It is likely that the different physical conditions caused by geographical environment and economic development of the NCMS enrollees may contributed to the differences in horizontal equity. Owning to the different eating habits, customs, topography and climate, there are slight differences in the health status of NCMS enrollees in these three regions. For example, Shanbei is located in the Loess Plateau, the precipitation is relatively less, only drought-tolerant crops could survive here, thus, miscellaneous grains are staple food here. As the Zhai DH & Tao LQ (2004) [36] highlighted that “Observing the effects of health, individuals who usually eat miscellaneous grain, fish, egg, pickled vegetables and tea have better health status than those who don’t”.

This study revealed that the health status of low-income groups was poor; however, their utilization rate of hospital services and inpatient benefits were low. The benefits of the NCMS were related directly to the utilization of medical services; the more they used medical services, the more benefits they could get. Although most rural residents participated in the NCMS, based on its current compensation policy, low-income enrollees were likely to abandon their use of hospital services due to the handicaps in paying for their own hospitalization needs. The NCMS funding model currently deploys residents’ quota financing, and it is then invested by the government to a certain percentage.

For the NCMS, the individual funding and government subsidy standards for different income groups were the same, but according to this payment method, the actual economic capacity of insured residents was not considered. Thereby, under the same circumstances, the inhibition of high-income groups was obviously weaker, helping rich enrollees benefit more. Prior analyses also confirmed this point of view; inequity was found to be in favour of the rich according to the inpatient benefit rates and compensation fees in the NCMS.

Our study has several strengths. Initially, current studies on the benefit equity of NCMS enrollees mainly use the concentration indices to measure the degree of inequity of benefits, but very few focus on the sources of inequity and horizontal inequity. We used the decomposition of the concentration index to analyse the sources of inequity and horizontal inequity, providing results and methods as a reference for improving relevant research.

This study also has several limitations that should be addressed in future studies. First, the hospital-related expenses and consumer spending data used in this study all were from the respondents themselves; thus, memory bias may be inevitable. Second, with the current data, we were unable to assess how psychological factors were related to inpatient benefits, as most factors in our study were related to physical conditions and socioeconomic characteristics. Third, we only studied the inpatient benefit rate and compensation fee because outpatient compensation is relatively limited in the NCMS. Thus the benefit rate and compensation in this study should be interpreted with caution, as our study mainly focused on NCMS enrollees in China, which may differ from those in other countries and those with different health insurance policies. Last, the fund of NCMS in Shaanxi province started to financed at city level in 2009 [37], the inpatient compensation standard is different in each city. Analyses of policy causes are beyond the capacity of our data for horizontal inequity. Policy implications are a good area for future inquiry by setting the research unit at the county or district level to create panel data.

## Conclusions

In conclusion, we found that there was inequity in favour of the rich in the inpatient benefit rate and compensation fee in the NCMS, which implies that wealthy enrollees benefit more. We also substantiated that the economic level, self-evaluated health scores and age contributed to the inequity of the inpatient benefit rate, while the economic level and age were the main contributing factors to the inequity of the inpatient compensation fee. Our findings underlined the need to strengthen the policies and programmes of the NCMS for the equity of basic health services, such as implementing hierarchical medical treatments to encourage patients’ primary diagnosis at the grassroots level. Additional inpatient benefits and compensation for the rich in high-level hospitals might need to be curbed to a relatively equitable level.

## Abbreviations

NCMS: The New Rural Cooperative Medical System
NHSS: National Health Service Survey
